# Spatial transcriptome analysis reveals Notch pathway-associated prognostic markers in *IDH1* wild-type glioblastoma involving the subventricular zone

**DOI:** 10.1186/s12916-016-0710-7

**Published:** 2016-10-26

**Authors:** Christine Jungk, Andreas Mock, Janina Exner, Christoph Geisenberger, Rolf Warta, David Capper, Amir Abdollahi, Sara Friauf, Bernd Lahrmann, Niels Grabe, Philipp Beckhove, Andreas von Deimling, Andreas Unterberg, Christel Herold-Mende

**Affiliations:** 1 Division of Experimental Neurosurgery, Department of Neurosurgery, Ruprecht-Karls-University Heidelberg, Im Neuenheimer Feld 400, 69120 Heidelberg, Germany; 2 Department of Neuropathology, Heidelberg University Hospital; CCU Neuropathology, German Consortium for Translational Cancer Research (DKTK), German Cancer Research Center (DKFZ), Heidelberg, Germany; 3 Department of Radiation Oncology, Heidelberg University Hospital; Molecular and Translational Radiation Oncology, National Center for Tumor Diseases (NCT), German Cancer Research Center (DKFZ), Heidelberg, Germany; 4 Hamamatsu Tissue and Imaging Analysis Center, University of Heidelberg, Heidelberg, Germany; 5 Division of Translational Immunology, German Cancer Research Center (DKFZ), Heidelberg, Germany; 6 Regensburg Center for Interventional Immunology (RCI), University Hospital, Regensburg, Germany

**Keywords:** Glioblastoma, Subventricular zone, mRNA microarray analysis, Location-dependent prognostic markers, Notch signaling

## Abstract

**Background:**

The spatial relationship of glioblastoma (GBM) to the subventricular zone (SVZ) is associated with inferior patient survival. However, the underlying molecular phenotype is largely unknown. We interrogated an SVZ-dependent transcriptome and potential location-specific prognostic markers.

**Methods:**

mRNA microarray data of a discovery set (*n* = 36 GBMs) were analyzed for SVZ-dependent gene expression and process networks using the MetaCore™ workflow. Differential gene expression was confirmed by qPCR in a validation set of 142 *IDH1* wild-type GBMs that was also used for survival analysis.

**Results:**

Microarray analysis revealed a transcriptome distinctive of SVZ+ GBM that was enriched for genes associated with Notch signaling. No overlap was found to The Cancer Genome Atlas’s molecular subtypes. Independent validation of SVZ-dependent expression confirmed four genes with simultaneous prognostic impact: overexpression of *HES4* (*p* = 0.034; HR 1.55) and *DLL3* (*p* = 0.017; HR 1.61) predicted inferior, and overexpression of *NTRK2* (*p* = 0.049; HR 0.66) and *PIR* (*p* = 0.025; HR 0.62) superior overall survival (OS). Additionally, overexpression of *DLL3* was predictive of shorter progression-free survival (PFS) (*p* = 0.043; HR 1.64). Multivariate analysis revealed overexpression of *HES4* to be independently associated with inferior OS (*p* = 0.033; HR 2.03), and overexpression of *DLL3* with inferior PFS (*p* = 0.046; HR 1.65).

**Conclusions:**

We identified four genes with SVZ-dependent expression and prognostic significance, among those *HES4* and *DLL3* as part of Notch signaling, suggesting further evaluation of location-tailored targeted therapies.

**Electronic supplementary material:**

The online version of this article (doi:10.1186/s12916-016-0710-7) contains supplementary material, which is available to authorized users.

## Background

Despite recent advances in multimodal treatment, de novo glioblastoma (GBM) World Health Organization (WHO) grade IV remains one of the most intractable human cancers, with a median survival of less than 15 months [1] and few long-time survivors [2]. Extensive efforts have been made to maximize the extent of resection (EOR) with simultaneous preservation of neurologic function and quality of life [3, 4]. At the same time, there are apparent advances in postoperative radiotherapy, chemotherapy, antiangiogenic therapy, immunotherapy, and targeted therapies [5, 6]. Nevertheless, recurrence occurs almost inevitably, in most cases adjacent to the resection cavity, leading to non-standardized salvage therapies and ultimately to death. Treatment failure has been attributed in part to the fact that GBM is not a monoclonal disease but is characterized by intra- and intertumoral heterogeneity, resulting in divergent clinical presentation and response to treatment. In this context, several molecular subtypes have been identified [7, 8] with distinct driver mutations, prognostic impact, and prediction of treatment response, including a glioma-CpG island methylator phenotype (G-CIMP) [9] which is highly dependent on the presence of mutations in the isocitrate dehydrogenase 1 (*IDH1*) gene and is associated with improved patient outcome [10]. Tumor location with respect to distinct brain regions reflects another important aspect of intertumoral heterogeneity. In particular, vicinity of de novo GBM to the subventricular zone (SVZ) lining the lateral ventricles, one of the persisting neurogenic regions in the adult human brain [11, 12], has been linked to inferior patient outcome [13] and a distinct growth pattern. Lim et al. initially reported a series of 53 de novo GBMs that were preoperatively classified by their vicinity to the SVZ into four groups. Group I consisted of GBMs with the contrast-enhancing (CE) lesion contacting the SVZ and infiltrating the cortex, group II of tumors contacting the SVZ but not involving the cortex, group III of GBMs not contacting the SVZ but involving the cortex, and group IV of tumors neither contacting the SVZ nor infiltrating the cortex [14]. Group I GBMs were most likely to be multifocal at first diagnosis and to recur distant from the resection cavity, while group IV GBMs were always solitary lesions with recurrences exclusively adjacent to the primary site. The authors concluded that GBMs with (SVZ+) and without (SVZ–) contact to the SVZ might arise from different cells of origin and that SVZ+ GBMs might reflect tumors with a high content of SVZ stem cells that have undergone malignant transformation, a hypothesis that has rarely been explored in detail. In one of the few studies addressing this issue so far, Kappadakunnel et al. failed to identify a stem cell-derived gene signature by means of a DNA microarray analysis of 47 GBMs classified according to their relationship to the SVZ [15]. In contrast, in a phylogenetic approach making use of intraoperative fluorescence-guided multiple sampling (FGMS) of human GBMs and their adjacent (fluorescent) SVZs, Piccirillo et al. were able to identify the SVZ as a reservoir of malignant precursor clones in the majority of tumors analyzed [16]. Accordingly, several clinical studies have provided evidence that targeting the ipsilateral SVZ by irradiation is associated with superior survival in patients with GBM [17, 18], especially in combination with gross total resection (GTR) [19], supporting the hypothesis that the SVZ plays a role in GBM formation and propagation.

Even though SVZ+ GBMs appear to be associated with a distinct clinical and radiographic behavior, little is known about the molecular phenotype underlying these characteristics and potential biomarkers linked to this particular tumor location. Therefore, the aim of this study was to identify a gene signature distinctive of de novo GBM in vicinity to the SVZ and to discover location-dependent genes with a potential prognostic impact. Noteworthy, validation of differential gene expression and prognostic relevance was performed in a confirmatory patient cohort restricted to *IDH* wild-type (*wt*) GBM, excluding the unique molecular and prognostic phenotype of *IDH* mutant (*mt*) GBM.

## Methods

### Clinical data

**Fig. 1:**
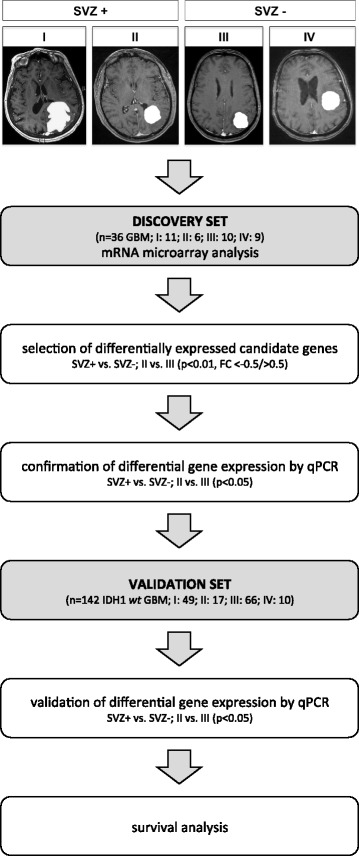
Flowchart illustrating the experimental design. De novo glioblastomas were allocated to different radiographic groups according to their vicinity to the SVZ as proposed by Lim et al. [14]. Group I consisted of contrast-enhancing tumors contacting the SVZ and infiltrating the cortex, group II of tumors contacting the SVZ only, group III of tumors contacting the cortex only, and group IV of tumors contacting neither SVZ nor cortex. Accordingly, groups I and II tumors were pooled as SVZ+ GBM, groups III and IV tumors as SVZ– GBM. Location-dependent differential gene expression was investigated by mRNA microarray analysis (*microarray cohort*; *n* = 36 GBM) and was validated by qPCR in a confirmatory patient sample (*validation cohort*; *n* = 142 *IDH1 wt* GBM) with subsequent survival analysis by log-rank test and multivariate Cox regression analysis

All demographic, treatment-related, and outcome data for patients with de novo GBM treated at the Department of Neurosurgery (University Hospital, Heidelberg, Germany) between 1998 and 2011 were obtained through review of medical charts and gathered in our institutional database. Approval from the ethics committee and written informed consent from patients were obtained in all cases and in accordance with the Declaration of Helsinki. EOR was determined for each patient on magnetic resonance imaging (MRI) scans taken within 72 hours post surgery and was deemed GTR if no residual contrast enhancement was detected; otherwise, EOR was classified as “subtotal” or “unknown” if no postoperative MRI was available. Radiographic classification of GBMs according to their vicinity to the SVZ was performed on preoperative contrast-enhanced T1-weighed MR images as described by Lim et al. [14] and depicted in Fig. 1. Group I consisted of tumors contacting the SVZ and infiltrating the cortex, group II of tumors contacting the SVZ only, group III of tumors contacting the cortex only, and group IV of tumors contacting neither SVZ nor cortex. Consequently, groups I and II tumors were pooled as SVZ+ GBM, and groups III and IV tumors as SVZ– GBM.

#### Microarray cohort

For microarray analysis, 36 patients with de novo GBM, typical radiographic presentation according to the classification proposed by Lim et al. [14], and availability of high-quality RNA (as described below) were investigated (group I: *n* = 11, group II: *n* = 6, group III: *n* = 10, group IV: *n* = 9). Median age at first diagnosis was 65 years; median overall survival (OS) and progression-free survival (PFS) were 11 and 3.5 months, respectively. All patients had died by July 2014. The rate of GTR was 31 %. *O* ^6^-methylguanine-DNA methyltransferase (MGMT) promoter hypermethylation was detected in 56 % of patients. *IDH1* mutation was present in one patient only (group III). There was no statistical difference regarding age distribution, EOR, survival, and molecular characteristics either between the four groups or when SVZ+ and SVZ– GBMs were compared (Table 1).

**Table 1:** Patient characteristics of the microarray cohort (*n* = 36 GBMs) and the validation cohort (*n* = 142 *IDH1 wt* GBMs), presented by location-dependent groups I–IV

Group	I	II	III	IV	Total	*p* value
Microarray cohort						
Patients, *n* (%)	11 (30)	6 (17)	10 (28)	9 (25)	36	
Sex, *n* (male : female)	5 : 6	6 : 0	7 : 3	5 : 4	23 : 13	0.14^a^
Age at diagnosis, mean (years)	68	64	59	64	65	0.21^b^
Extent of resection, *n* (%)						0.85^a^
- Complete	3 (27)	2 (33)	4 (40)	2 (22)	11 (31)	
- Partial	4 (36.5)	4 (67)	5 (50)	5 (56)	18 (50)	
- Unknown	4 (36.5)	0	1 (10)	2 (22)	7 (19)	
OS, median (months)	9	9.5	15	16	11.5	0.98^c^
PFS, median (months)	3	5.5	3	5	3.5	0.47^c^
Multifocal CE lesion, *n* (%)	2 (18)	0 (0)	1 (10)	0 (0)	3 (8)	0.42^a^
Multifocal FLAIR lesion, *n* (%)	2 (18)	0 (0)	3 (30)	0 (0)	5 (14)	0.19^a^
MGMT promoter methylation, *n* (%)						0.06^a^
- Yes	9 (82)	1 (17)	6 (60)	4 (44)	20 (56)	
- No	2 (18)	5 (83)	4 (40)	5 (56)	16 (44)	
*IDH1* mutation, *n* (%)						0.44^a^
- Yes	0	0	1 (10)	0	1 (3)	
- No	11 (100)	6 (100)	9 (90)	9 (100)	35 (97)	
Validation cohort						
Patients, *n* (%)	49 (35)	17 (12)	66 (46)	10 (7)	142	
Sex, *n* (male : female)	31 : 18	9 : 8	41 : 25	6 : 4	87 : 55	0.89^a^
Age at diagnosis, mean (years)	62	57	62	64	62	0.32^d^
KPS preoperative, mean	78	76	86	86	82	**0.01** ^**d**^
Extent of resection, *n* (%)						**0.006** ^**a**^
- Complete	8 (16)	6 (35)	23 (35)	7 (70)	44 (31)	
- Partial	37 (76)	9 (53)	31 (47)	2 (20)	79 (56)	
- Unknown	4 (8)	2 (12)	12 (18)	1 (10)	19 (13)	
Radiation therapy, *n* (%)	43 (88)	16 (94)	62 (94)	8 (80)	129 (91)	0.4^a^
Temozolomide, *n* (%)	30 (61)	14 (82)	43 (65)	8 (80)	95 (67)	0.33^a^
OS, median (months)	12	14	15	17	13	**0.004** ^**c**^
PFS, median (months)	6	8	7	8	7	0.27^c^
Censored patients, *n* (%)	2 (4)	1 (6)	4 (6)	0 (0)	7 (5)	0.85^a^
MGMT promoter methylation						0.43^a^
- Yes, *n* (%)	10 (20)	7 (41)	17 (26)	4 (40)	38 (26)	
- No, *n* (%)	16 (33)	6 (35)	27 (41)	3 (30)	52 (37)	
- Unknown, *n* (%)	23 (47)	4 (24)	22 (33)	3 (30)	52 (37)	
*IDH1* mutation, *n* (%)						N/A
- No, *n* (%)	49 (100)	17 (100)	66 (100)	10 (100)	142(100)	

*OS* overall survival, *PFS* progression-free survival, *CE* contrast-enhancing, *FLAIR* fluid attenuated inversion recovery, *MGMT O* ^6^-methylguanine-DNA methyltransferase, *IDH1* isocitrate dehydrogenase 1, *KPS* Karnofsky performance score

^a^Chi-square test

^b^Kruskal-Wallis test

^c^Log-rank test

^d^One-way ANOVA

N/A not applicable

In bold: *p* < 0.01

#### Validation cohort

For independent validation of microarray expression data and evaluation of a prognostic significance of single candidate genes, a validation set of 142 patients with *IDH1 wt* GBM was analyzed (Table 1) for whom radiographic classification was conducted as well. Median age at first diagnosis was 62 years and median preoperative Karnofsky performance score (KPS) was 82 %. Of these patients, 31 % received GTR and the majority postoperative radiation therapy (91 %). Temozolomide (TMZ) was administered in two-thirds of patients. Median OS was 13 months and median PFS was 7 months. Seven patients were alive at the end of the study (July 2014) and thus censored for survival analysis. MGMT promoter hypermethylation was present in 26 %, absent in 37 %, and not available in 37 % of patients.

### Patient material, quality control, and RNA extraction

Tumor tissue was obtained following surgical resection at the Department of Neurosurgery (University Hospital, Heidelberg, Germany), immediately snap-frozen, and stored at –80 °C until further processing. Due to the retrospective nature of this study, the exact sampling position with regard to distance to the SVZ was not determinable; tumors were rather allocated to one of the four location groups based on their radiographic appearance. Two board-certified neuropathologists confirmed histopathological diagnosis and quality control regarding tumor content (>60 %) and necrosis (<20 %). Comparing the distribution of tumor content between the four location groups did not reveal a significant difference (Additional file 1: Figure S1A). To ensure that differential gene expression in mRNA microarray analysis was not affected by location-specific differences in tumor microenvironment, we applied the ESTIMATE algorithm from Yoshihara et al. [20], as described in detail in Additional file 1: Figure S1B–D and Additional file 2. *IDH1* mutation and MGMT promoter methylation status were determined as described elsewhere [2, 21, 22]. RNA was extracted with the AllPrep® DNA/RNA/Protein mini kit (Quiagen, Hilden, Germany) according to the manufacturer’s instructions from high-quality tissue samples. Analyte concentration and quality were determined using a Nanodrop 2000 spectrophotometer (Thermo Scientific) and a Bioanalyzer 2100 (Agilent), respectively.

### Processing of microarray data

1 μg total RNA from 36 GBM tissues was submitted to the Genomics Core Facilities of the German Cancer Research Center (DKFZ, Heidelberg, Germany) for microarray analysis. After purification, reverse transcription to cDNA, and labeling according to the Illumina protocol [23], samples were hybridized to Human HT-12 v.4.0 arrays (Illumina). Raw-intensity data were obtained after image analysis of the fluorescent spot intensity reads. All preprocessing and normalization steps were performed in the *R* programming environment [www.r-project.org]. Interarray normalization was conducted using *qspline* normalization in the *affy* package [24, 25]. After median probe set summarization, a linear model was fitted to account for different batches (*limma* package). Lastly, intraarray normalization was performed by median centering of the data, followed by log2 transformation. Data were deposited at the NCBI Gene Expression Omnibus [GEO:GSE83537].

### Assessment of molecular subtypes in microarray cohort

Centroids established by Verhaak et al. [8] for subtyping of GBM expression data were downloaded from The Cancer Genome Atlas (TCGA) working group website (the accompanying data freeze was released with the aforementioned publication). For each case, correlation (Pearson’s *r*) between respective expression values and centroids was calculated for all genes available in the data set (*n* = 800 out of 840). Subsequently, each sample was assigned the subtype of the centroid with which it was most strongly correlated.

### Real-time PCR

Quantitative PCR (qPCR) was performed to confirm mRNA microarray expression data and differential expression of single candidate genes in the validation cohort. Primer design and selection of corresponding hybridization probes was done using the Universal ProbeLibrary Assay Design Center (http://lifescience.roche.com). Primers were obtained from Sigma-Aldrich (St. Louis, MO, USA) and along with the probes are summarized in Additional file 3: Table S1. RT-PCR reactions were performed according to the manufacturer’s instructions using 45 amplification cycles (LightCycler LC480, cDNA First Strand Transcriptor Kit, LightCycler TaqMan Master, Universal ProbeLibrary Set (human); all Roche Diagnostics, Mannheim, Germany). Quantification of mRNA expression was performed in triplicate and referenced to a set of housekeeping genes: glyceraldehyde-3-phosphate dehydrogenase (GAPDH), beta-actin (ACTB), and hypoxanthine-guanine phosphoribosyltransferase 1 (HPRT1). Only triplicates with a deviation in crossing point (Cp) values of less than <0.55 were deemed appropriate for further relative quantification employing *qbase +* software version 2.5 (Biogazelle NV, Zwijnaarde, Belgium).

### Statistical analyses

Unless stated otherwise, statistical analyses were conducted in *R* (www.r-project.org). Differential gene expression in GBM subgroups was assessed using a two-sided Student’s *t* test and a Mann-Whitney test as indicated. For survival analysis, PFS and OS were used as end points. PFS was defined as the time interval from first histologic diagnosis to radiologic signs of progression/recurrence or death, whatever occurred first. OS was defined as the time interval from first histologic diagnosis until death or last follow-up. Prognostic significance was determined using univariate and multivariate Cox regression analyses and log-rank tests. For multivariate models, all clinico-pathological parameters significant in the univariate analysis were included. Enrichment analysis for process networks was performed using the MetaCore™ analysis workflow.

## Results

### SVZ-dependent transcriptional profiles in de novo GBM

**Fig. 2:**
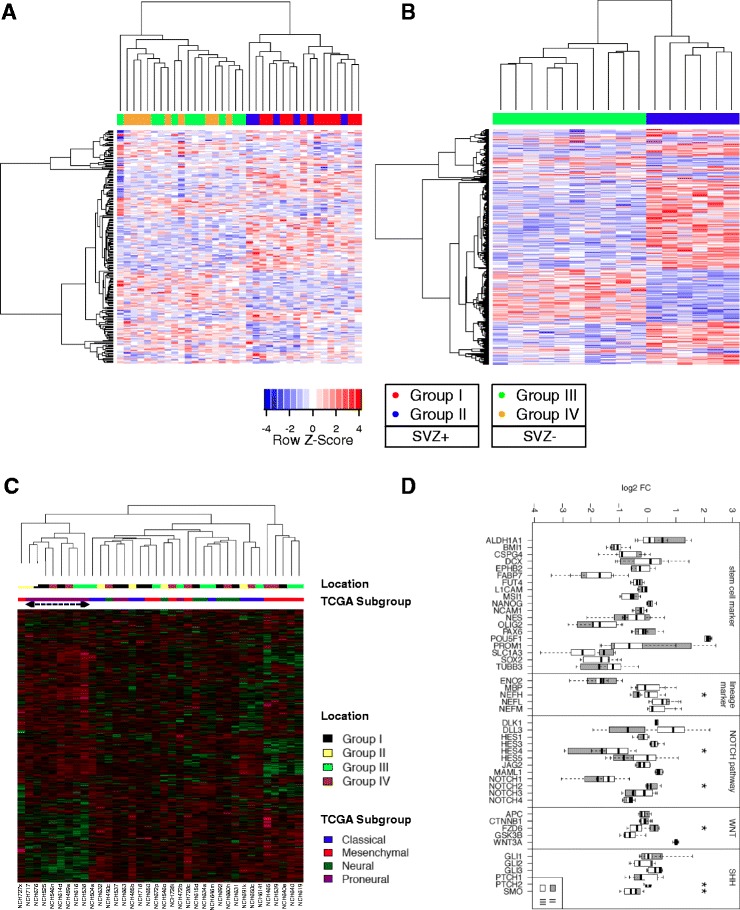
**a**, **b** Hierarchical clustering of mRNA microarray data revealed two main transcriptional profiles that reflect the allocation of tumors according to their vicinity to the SVZ in general (**a**; top 222 genes differentially expressed between SVZ+ and SVZ– GBMs (*p* < 0.01)) and to group II (GBMs contacting the SVZ only) and group III (GBMs contacting the cortex only) tumors in particular (**b**; top 312 genes; *p* < 0.01). **c** There was no overlap between location-dependent (SVZ+/–) gene signatures and the molecular subtypes reported by the TCGA working group. **d** Box plots depicting differential expression of genes considered as stem cell markers, lineage markers, and markers of three developmental pathways (Notch, Wnt, Sonic Hedgehog (SHH)) as identified by mRNA microarray analysis. Note that significant differences (*p* < 0.05; marked with asterisk) were observed for a subset of genes only (*FZD6*, *PTCH2*, *NOTCH2*, *HES4*, *NEFH*) together with a strong trend for *DLL3* (*p* = 0.0654), notably including three genes with involvement of Notch signaling

First, an mRNA microarray analysis was performed for a discovery set of 36 de novo GBMs (*microarray cohort*) allocated to one of the four radiographic groups proposed by Lim et al. [14]. Hierarchical clustering of the top 222 genes differentially expressed between SVZ+ and SVZ– GBMs (*p* < 0.01) revealed distinct transcriptional profiles that perfectly discriminated between these two groups (Fig. 2a). This effect was most pronounced when only the two most contrary GBM groups (group II (contacting SVZ only) and group III (contacting cortex only)) that separate best between GBMs with and without SVZ involvement were compared (top 312 genes; *p* < 0.01) (Fig. 2b). To learn more about the functional significance of these gene signatures, a MetaCore™ enrichment analysis was performed to identify the top 10 pathways associated with differential gene expression. Enrichment analysis of the top 1494 genes differentially expressed between SVZ+ and SVZ– GBMs (*p* < 0.05) revealed upregulation of genes linked to chromatin modification and downregulation of genes linked to Notch signaling, blood vessel morphogenesis, and immune modulation (T cell receptor signaling, interleukin-2 (IL-2) signaling, leukocyte chemotaxis) in SVZ+ GBMs (Table 2). Comparing the top 1573 genes differentially expressed between group II and group III GBMs (*p* < 0.05), genes related to neurogenesis were upregulated, and genes related to blood vessel morphogenesis and immune modulation (IL-2 signaling, leukocyte chemotaxis) were downregulated in group II GBMs contacting the SVZ only (Table 3). We further explored a potential overlap between SVZ-dependent transcriptomes and the molecular subtypes reported by the TCGA working group [8] but found none (Fig. 2c).

**Table 2:** Summary of enrichment analysis for the top 1494 differentially expressed genes between groups I, II (SVZ+) versus III, IV (SVZ–) (*p* < 0.05)

MetaCore^TM^ process network	Upregulated in SVZ+ (*n* = 771)	Downregulated in SVZ+ (*n* = 723)
Term	*p* value (*n*_upregulated_/*n*_total_)	*p* value (*n*_downregulated_/*n*_total_)
Blood vessel morphogenesis	0.669 (4/228)	**1.55E-04 (16/228)**
Regulation of epithelial-to-mesenchymal transition	0.943 (2/226)	**4.57E-04 (15/226)**
Leukocyte chemotaxis	0.985 (1/205)	**5.32E-04 (14/205)**
Chromatin modification	**9.61E-04 (9/128)**	0.392 (4/128)
T cell receptor signaling	0.971 (1/174)	**1.22E-03 (12/174)**
Cytoskeleton - actin filaments	0.465 (4/176)	**1.35E-03 (12/176)**
Notch signaling	0.507 (5/236)	**2.08E-03 (14/236)** 0.480 (4/144)
Skeletal muscle development	**2.19E-03 (9/144)**	
Cell cycle G1/S	0.407 (4/163)	**2.31E-03 (11/163)**
Inflammation - IL-2 signaling	0.878 (1/104)	**4.11E-03 (8/104)**

The top 10 enrichments according to *p* value are depicted (in bold: *p* < 0.01). In addition, the total number of genes per process network (*n*_total_) as well as the intersect with significantly up- or downregulated genes are displayed (*n*_upregulated_ and *n*_downregulated_)

**Table 3:** Summary of enrichment analysis for the top 1573 differentially expressed genes between groups II and III (*p* < 0.05)

MetaCore^TM^ process network	Upregulated in group II (*n* = 957)	Downregulated in group II (*n* = 616)
Term	*p* value (*n*_upregulated_/*n*_total_)	*p* value (*n*_downregulated_/*n*_total_)
Blood vessel morphogenesis	0.058 (10/228)	**2.14E-07 (20/228)**
Muscle contraction	**1.22E-03 (12/173)**	0.411 (7/173)
Male sex differentiation	0.087 (10/246)	**1.50E-03 (14/246)**
Transmission of nerve impulse	**2.36E-03 (13/212)**	0.360 (6/212)
Neurogenesis	**2.95E-03 (12/192)**	0.452 (5/192)
Chemotaxis	0.669 (3/137)	**4.13E-03 (9/137)**
Anti-apoptosis via PI3K/Akt	0.982 (2/233)	**7.16E-03 (12/233)**
Leukocyte chemotaxis	0.891 (3/205)	**7.36E-03 (11/205)**
Neurohormone signaling	0.273 (7/211)	**9.06E-03 (11/211)**
Inflammation - IL-2 signaling	0.482 (3/104)	**9.73E-03 (7/104)**

The top 10 enrichments according to *p* value are depicted (in bold: *p* < 0.01). In addition, the total number of genes per process network (*n*_total_) as well as the intersect with significantly up- or downregulated genes are displayed (*n*_upregulated_ and *n*_downregulated_)

*PI3K* phosphatidylinositide 3-kinase, *Akt* protein kinase B

### Identification of candidate genes distinctive of SVZ+ GBM

To identify single candidate genes with location-dependent differential expression, mRNA microarray data were further compared between SVZ+ and SVZ– GBMs and group II and group III GBMs, respectively. Candidate genes had to meet the following criteria: a *p* value (two-sided *t* test) of <0.01 and/or a log2 fold change (FC) of >0.5/<–0.5 in at least one of the two comparisons (Fig. 1). Thus, 26 genes were identified (Table 4). To exclude false positive results, qPCR analysis of mRNA expression was performed for all tumor samples of the microarray cohort. Differential gene expression on a *p* < 0.05 level (two-sided Mann-Whitney test) was verified for 16 genes: *PIR* (pirin), *HES4* (hairy and enhancer of split 4), *DLL3* (delta-like 3), *NTRK2* (neurotrophic receptor tyrosine kinase type 2), *IGFBP5* (insulin-like growth factor-binding protein 5), *BAI3* (brain-specific angiogenesis inhibitor 3), *EMILIN-3* (elastin microfibril interfacer 3), *FERMT2* (fermitin family member 2), *CDH4* (cadherin 4), *HIF1A* (hypoxia inducible factor 1, alpha subunit), *RBP1* (retinol binding protein 1), *SYTL4* (synaptotagmin-like 4), *THBS4* (thrombospondin 4), *FZD6* (frizzled class receptor 6), *ENPP5* (ectonucleotide pyrophosphatase/phosphodiesterase 5), and *BATF3* (basic leucine zipper transcription factor, ATF-like 3). See Table 4. Two of these genes (*EMILIN-3* and *CDH4*) have never been reported in the context of glioma research so far. *BATF3* had to be excluded from further analysis in the validation cohort due to a negative correlation of expression data, and *FZD6* and *ENPP5* due to a high variance of expression values. All other genes (*n* = 13) were subjected to an independent validation of differential gene expression by means of qPCR in the validation cohort (*n* = 142 patients with *IDH1 wt* GBM).

**Table 4:** Tabular summary of SVZ-dependent expression of genes

Gene	Microarray cohort (*n* = 36)						Selected for validation	Validation cohort (*n* = 142)		
	SVZ+ vs. SVZ–			II vs. III				SVZ+ vs. SVZ–	II vs. III	Expression in SVZ+/II
	*p* value	FC	qPCR	*p* value	FC	qPCR		*p* value	*p* value	
***PIR***	0.0188	0.4770	**0.0082**	**0.0047**	**0.7338**	**0.0225**	✓	0.1597	**0.05**	Low
***HES4***	0.3680	−0.0135	**0.0015**	0.0440	**−0.5945**	0.5584	✓	**0.0102**	**0.0007**	High
***DLL3***	0.5645	**−0.7725**	0.1407	0.0654	**−1.5897**	**0.0295**	✓	0.9516	0.0857	High (trend)
***NTRK2***	0.0511	0.2464	0.5821	0.0304	**0.5708**	0.0559	✓	0.7883	0.0558	High (trend)
***IGFBP5***	0.0134	0.4772	**0.0066**	0.0234	**1.2149**	0.1179	✓	0.8477	0.9498	–
***BAI3***	0.3676	−0.0817	0.1829	0.0290	**−0.5699**	0.0714	✓	0.2911	0.8653	
***EMILIN3***	0.0810	0.3924	0.3921	0.0290	**0.7203**	**0.0312**	✓	0.6789	0.5567	
***FERMT2***	0.0109	−0.1693	**0.0058**	**0.0088**	**−0.8139**	**0.0017**	✓	0.7765	0.1156	
***CDH4***	0.0197	−0.3874	**0.0168**	0.0929	**−0.7704**	**0.0120**	✓	0.2006	0.7516	
*UCHL1*	0.0168	0.3306	0.1369	0.2702	0.1823	0.7063				
***HIF1A***	**0.0052**	−0.1433	**0.0215**	0.1274	−0.1174	0.5345	✓	0.6371	0.7699	
*TGFB3*	**0.0021**	**−0.5547**	0.2056	0.0570	**−0.6072**	0.3000				
***RBP1***	**0.0017**	**0.6986**	**0.0186**	**0.0035**	**1.2351**	**0.0312**	✓	0.9776	0.6936	
***SYTL4***	0.1318	0.0785	0.2347	**0.0035**	**1.0968**	**0.0075**	✓	0.3189	0.4702	
***THBS4***	0.0155	0.1638	0.1810	**0.0096**	**0.8009**	**0.0072**	✓	0.4495	0.9817	
*NODAL*	**0.0043**	−0.4242	0.3117	0.0225	−0.4279	0.2625				
***FZD6***	0.1895	0.1517	0.4073	**0.0014**	**0.6362**	0.0730				
*VAV3*	0.0873	0.1032	0.5665	0.0235	**0.8138**	0.4895				
***ENPP5***	0.3551	0.2665	**0.0397**	0.0218	**0.6382**	**0.0116**				
*TIMP4*	0.4696	0.1804	0.1286	0.0417	**0.5615**	0.1179				
*NDN*	0.0107	**0.8016**	0.2038	0.2543	**1.3415**	0.9458				
*NFKBIA*	0.0149	−0.1440	0.3924	0.0144	−0.3358	0.2515				
*IRF9*	0.0750	−0.0766	0.2604	0.0157	**−0.5397**	0.2630				
***BATF3***	0.1257	−0.1263	**0.0414**	**0.0087**	**−0.6015**	0.6570				
*ANGPTL2*	0.2385	−0.1070	0.5178	0.0187	**−0.6711**	0.7632				
*PDGFRA*	0.9394	−0.0442	0.2959	0.0453	**−1.1161**	0.0714				

First, mRNA microarray data were compared between SVZ+ and SVZ– GBM and group II and group III GBM, respectively (in bold: *p* < 0.01 (two-sided *t* test), log2 fold change (*FC*) >0.5/<–0.5) with technical validation of differential gene expression by qPCR (in bold: *p* < 0.05 (two-sided Mann-Whitney test)) (microarray cohort). Based on the prespecified significance levels, 13 candidate genes (indicated by check marks) were chosen for validation of differential gene expression by qPCR (in bold: *p* < 0.05 (two-sided Mann-Whitney test)) in a confirmatory patient cohort (validation cohort)

**Fig. 3:**
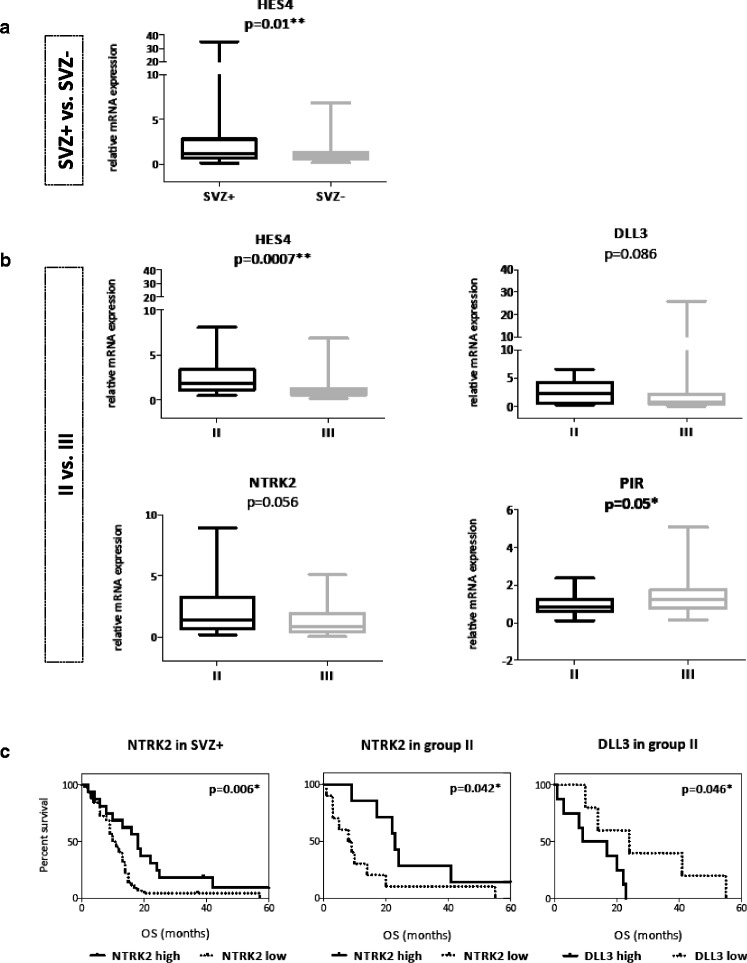
**a**, **b** Box plots illustrating differential expression of candidate genes in the validation cohort as confirmed by qPCR. **a** Significant overexpression of *HES4* in SVZ+ GBMs. **b** Significant overexpression of *HES4* in group II GBMs. Also, there was a strong trend towards overexpression of *DLL3* and *NTRK2* in group II GBMs and *PIR* in group III GBMs. **c** Location-dependent prognostic significance of *NTRK2* in SVZ+ GBMs (*left panel*) as well as *NTRK2* (*middle panel*) and *DLL3* (*right panel*) in group II GBMs. Superior OS was observed in patients with ≥75 % expression of *NTRK2* (Q1 cut-off) and <50 % expression of *DLL3* (median cut-off), respectively

As expected, independent validation of location-dependent differential expression was accomplished for a subset of genes only. In SVZ+ GBMs, upregulation of *HES4* (*p* = 0.01) was observed, a finding that was even more pronounced (*p* = 0.0007) when group II GBMs (contacting the SVZ only) were compared to group III GBMs (contacting the cortex only). In group II GBMs, there was also a strong trend for upregulation of *DLL3* (*p* = 0.086) and *NTRK2* (*p* = 0.056) and downregulation of *PIR* (*p* = 0.05) (Table 4; Fig. 3a, b).

### SVZ-dependent regulation of genes involved in Notch signaling

**Fig. 4:**
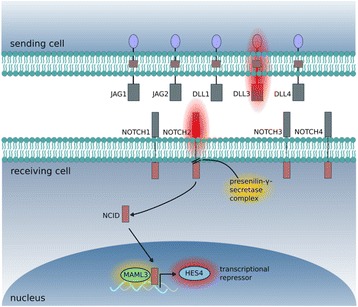
mRNA microarray analysis (*microarray cohort*; *n* = 36 GBMs) revealed SVZ-dependent differential gene expression of key hierarchies of the Notch pathway. Results are visualized comparing group II (contacting the SVZ only) and group III (involving the cortex only) GBMs, since this grouping discriminates most precisely between GBMs with and without SVZ involvement. In group II GBMs, significant overexpression (*p* < 0.05; highlighted in *red*) was observed for *DLL3* (ligand to Notch receptors), *NOTCH2* (Notch transmembrane receptor), and *HES4* (nuclear target gene to Notch signaling; tissue-specific transcription repressor). For *PSEN2* (presenilin2) and *NCSTN* (nicastrin), two components of the presenilin-gamma-secretase complex that is involved in cleavage of the intracellular Notch receptor domain, and *MAML3* (mastermind like transcriptional coactivator 3; amplifies Notch-induced transcription), overexpression did not reach significance (highlighted in *yellow*)

It has been hypothesized that de novo GBMs with and without contact to the SVZ are derived from different cells of origin with SVZ+ GBMs enriched for (cancer) stem cells [11, 12, 14]. In this study, MetaCore™ pathway analysis of mRNA microarray data did not reveal enrichment in classical (cancer) stem cell pathways except for a downregulation of genes linked to Notch signaling in SVZ+ GBMs (Table 2). When comparing the two most contrary location-specific groups II and III by means of MetaCore™, Notch signaling was not among the top signaling networks, but group II GBMs were enriched for genes related to neurogenesis (Table 3). Keep in mind, however, that a significant *p* value for the enrichment analysis does not necessarily imply a meaningful down- or upregulation of the pathway, but a mere enrichment of the differentially upregulated or downregulated genes for the respective process network (Tables 2 and 3). As enrichment analyses with a curated gene list or process networks can only serve as a starting point for further analysis, we decided to hand-search our mRNA microarray data for an additional selection of 47 published markers of neural stem cells, radial glia cells, and brain tumor-initiating cells (BTICs), lineage markers and key players of three developmental pathways (Notch, Wnt, Sonic Hedgehog) in view of a potential location-dependent difference in gene expression. In this regard, we chose to compare groups II and III GBMs since this represents the most distinct separation between GBMs with (group II) and without (group III) contact to the SVZ (Fig. 2d). However, an unequivocal SVZ-dependent (cancer) stem cell signature was not detected. In fact, differential gene expression was observed for a subset of genes only (*FZD6*, *NOTCH2*, *PTCH2* and *HES4*, *DLL3*, *NEFH*, respectively). Notably, three of these genes (*DLL3*, *NOTCH2*, *HES4*) are involved in Notch signaling: *DLL3* is a ligand to Notch receptors (among those *NOTCH2*), while *HES4* is a target gene of Notch signaling that acts as a tissue-specific repressor (Fig. 4). This observation prompted us to search our microarray data for further components of the Notch pathway. Indeed, a non-significant differential regulation was found for *PSEN2* (presenilin2) and *NCSTN* (nicastrin), two genes involved in the intracellular cleavage of Notch receptors, and for *MAML3* (mastermind like transcriptional coactivator 3) that amplifies Notch-induced transcription (Fig. 4). For *HES4* and *DLL3*, SVZ-dependent differential gene expression was confirmed in further analyses of the validation cohort (Table 4; Fig. 3a, b), along with a significant prognostic impact on patient outcome (Table 5), as described below.

**Table 5:** Clinical and molecular prognostic factors of overall (OS) and progression-free (PFS) survival in the validation cohort (*n* = 142 *IDH wt* patients) based on a univariate log-rank test and a multivariate Cox regression model

		OS		PFS	
		HR (95 % CI)	*p* value	HR (95 % CI)	*p* value
Univariate					
Clinico-pathological parameters					
Age at first diagnosis		1.05 (1.03–1.07)	**4.0E-07**	1.02 (1.00–1.04)	**0.020**
Gender (male)		0.99 (0.70–1.41)	0.971	1.33 (0.87–2.04)	0.187
Radiotherapy		0.29 (0.16–0.52)	**9.7E-06**	0.99 (0.31–3.13)	0.983
Chemotherapy		0.41 (0.28–0.59)	**1.4E-06**	0.58 (0.37–0.91)	**0.017**
Preoperative KPS		0.98 (0.97–0.99)	**0.002**	1.00 (0.99–1.01)	0.810
MGMT methylation		0.83 (0.53–1.29)	0.401	1.15 (0.69–1.92)	0.588
GTR		0.42 (0.28–0.62)	**8.8E-06**	0.78 (0.49–1.23)	0.286
Location (SVZ+)		1.39 (0.99–1.96)	0.056	1.07 (0.71–1.61)	0.743
Location (II vs. III)		1.19 (0.69–2.08)	0.526	1.30 (0.66–2.60)	0.445
Candidate genes					
	Cut-offs				
*IGFBP5*	Q1	1.62 (1.09–2.39)	**0.015**	1.19 (0.73–1.94)	0.475
*DLL3*	Median	1.61 (1.08–2.39)	**0.017**	1.64 (1.01–2.68)	**0.043**
*NTRK2*	Q1	0.66 (0.44–1.00)	**0.049**	0.64 (0.39–1.07)	0.086
*PIR*	Q1	0.62 (0.41–0.95)	**0.025**	0.66 (0.40–1.08)	0.092
*HES4*	Q3	1.55 (1.03–2.32)	**0.034**	1.31 (0.81–2.13)	0.268
Multivariate					
Clinico-pathological parameters					
Age at first diagnosis		1.03 (1.00–1.06)	**0.024**	1.01 (0.99–1.04)	0.198
Radiotherapy		0.22 (0.08–0.58)	**0.002**		
Chemotherapy		0.55 (0.29–1.05)	0.072	0.62 (0.36–1.06)	0.082
Preoperative KPS		0.99 (0.98–1.01)	0.504		
GTR		0.48 (0.29–0.83)	**0.007**		
Location (SVZ+)		1.82 (1.09–3.04)	**0.023**		
Candidate genes					
	Cut-offs				
*IGFBP5*	Q1	0.82 (0.48–1.42)	0.488		
*DLL3*	Median	1.47 (0.83–2.60)	0.181	1.65 (1.00–2.70)	**0.046**
*NTRK2*	Q1	0.75 (0.41–1.38)	0.358		
*PIR*	Q1	0.75 (0.41–1.36)	0.344		
*HES4*	Q3	2.03 (1.06–3.90)	**0.033**		

Gene expression data were dichotomized according to the median in “high” and “low” expression (“median cut-off”) and according to quartiles either in “top 25 % expression” and “<75 % expression” (“Q1 cut-off”) or “bottom 25 % expression” and “>25 % expression” (“Q3 cut-off”). *p* values < 0.05 are displayed in bold letters

*HR* hazard ratio, *CI* confidence interval, *KPS* Karnofsky performance score, *MGMT O* ^6^-methylguanine-DNA methytransferase, *GTR* gross total resection

### Identification of prognostic markers distinctive of SVZ+ GBM

In our validation cohort of 142 patients with *IDH wt* GBM, age at first diagnosis, radiotherapy, adjuvant chemotherapy, preoperative KPS and GTR, together with a clear trend for SVZ involvement (*p* = 0.056; HR 1.39 (0.99–1.96)), were predictive for OS, while age at first diagnosis and adjuvant chemotherapy were predictive for PFS (Table 5). In the multivariate analysis, vicinity to the SVZ (SVZ+ GBM) was an independent prognosticator of inferior OS (*p* = 0.023; HR 1.82 (1.09–3.04); 12 versus 15 months) but did not affect PFS (Table 5). As expected from the literature, GTR was an independent prognosticator of superior OS (*p* = 0.007; HR 0.48 (0.29–0.83)). Note that the rate of GTR was significantly higher in SVZ– GBM compared to SVZ+ GBM (39 % versus 21 %; *p* = 0.019; Fisher’s exact test). Radiotherapy (*p* = 0.002; HR 0.22 (0.08–0.58)) and age at first diagnosis (*p* = 0.024; HR 1.03 (1.00–1.06)) were also associated with OS in the multivariate analysis.

Next, we asked if our location-specific candidate genes discovered by microarray analysis (*HES4*, *DLL3*, *NTRK2*, *PIR*) conferred a prognostic impact; therefore, we investigated a possible association between mRNA expression levels and patient outcome in the validation cohort. Since nothing is known about the biological power of candidate gene expression levels, expression data were dichotomized both according to the median in “high” and “low” expression (“median cut-off”) and according to quartiles either in “top 25 % expression” and “<75 % expression” (“Q1 cut-off”) or “bottom 25 % expression” and “>25 % expression” (“Q3 cut-off”). Univariate analysis revealed a prognostic impact for all of these genes. High expression of *HES4* (*p* = 0.034, HR 1.55 (1.03–2.32); Q3 cut-off) and *DLL3* (*p* = 0.017, HR 1.61 (1.08–2.39); median cut-off) predicted inferior OS, while high expression of *NTRK2* (*p* = 0.049, HR 0.66 (0.44–1.00); Q1 cut-off) and *PIR* (*p* = 0.025, HR 0.62 (0.41–0.95); Q1 cut-off) predicted superior OS. High expression of *DLL3* was also predictive of inferior PFS (*p* = 0.043, HR 1.64 (1.01–2.68); median cut-off). See Table 5. Also, a negative prognostic impact on OS was revealed for *IGFBP5* (*p* = 0.015, HR 1.62 (1.09–2.39); Q1 cut-off), one of the candidate genes for which location-dependent gene expression could not be confirmed in the validation cohort. Note that *NTRK2* and *DLL3* were identified as location-specific prognostic markers: in SVZ+ GBMs, OS was significantly prolonged in patients with high *NTRK2* expression levels (*p* = 0.006; Q1 cut-off; 18.5 versus 10.5 months). In group II GBMs, a significantly higher OS was observed in patients with high *NTRK2* expression (*p* = 0.042; Q1 cut-off; 23 versus 8.5 months) and with low *DLL3* expression (*p* = 0.046; median cut-off; 24 versus 13 months), respectively (Fig. 3c). Most importantly, multivariate survival analysis revealed a negative prognostic impact of *HES4* on OS (*p* = 0.033; HR 2.03 (1.06–3.9)), independent of all other candidate genes and clinical factors with significant survival impact in univariate analysis, and of *DLL3* on PFS (*p* = 0.046; HR 1.65 (1.00–2.70)). See Table 5.

**Fig. 5:**
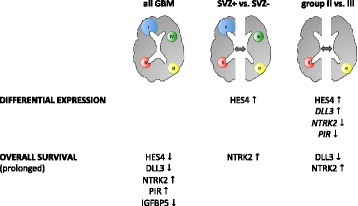
Graphical summary of location-dependent differential gene expression and identification of prognostic markers, comparing SVZ+ (groups I and II) and SVZ– (groups III and IV) GBMs and group II (contacting the SVZ only) and group III (involving the cortex only) GBMs, respectively

Taken together, our stepwise approach identified four genes (*HES4*, *DLL3*, *PIR*, *NTRK2*) with SVZ-specific expression and simultaneous prognostic significance (summarized in Fig. 5). In multivariate survival analysis, *HES4* was an independent prognosticator of OS and *DLL3* of PFS. Both overall pathway analysis and in-depth analysis of single candidate genes point to a relevant involvement of Notch signaling in SVZ+ GBM.

## Discussion

Intertumoral heterogeneity is one of the mainstays of treatment failure in GMB; thus, there is a need for individualized prognostication and treatment approaches. Tumor location is one important aspect that clearly determines treatment options, functional outcome, and quality of life. However, it is largely unknown whether tumor location is linked to a distinct molecular phenotype. In this study we sought to compare transcriptomes of GBMs with and without spatial relationship to the SVZ in order to identify location-dependent gene signatures and prognostic markers. In contrast to previous studies, location-dependent candidate genes identified in a discovery set were validated in an independent patient cohort comprising exclusively *IDH wt* GBM. Thereby, we sought to exclude the unique molecular and prognostic phenotype of *IDH mt* GBM [9].

Hierarchical clustering of microarray data revealed two main transcriptional profiles that perfectly matched the allocation of tumors according to their vicinity to the SVZ in general and to group II (GBMs contacting the SVZ only) and group III (GBMs contacting the cortex only) tumors in particular. MetaCore™ enrichment analysis linked these SVZ-dependent transcriptomes to major pathways involved in cell growth and motility, angiogenesis, immune modulation, and Notch signaling, one of the major developmental pathways involved in neural stem cell (NSC) maintenance and gliomagenesis [26, 27]. Importantly, no overlap was found between our location-specific transcriptional profiles and the four molecular subgroups described by the TCGA [8]. In face of our study’s relatively small case number, tumor vicinity to the SVZ does not appear as a determining factor of the TCGA’s molecular phenotypes.

Ever since the early reports that vicinity of GBM to the SVZ is linked to a distinct growth behavior and inferior patient outcome [13–15], it has been hypothesized that SVZ+ GBM may arise from transformed NSCs originally residing within the SVZ [11, 12]. Indeed there is evidence from rodent studies that inactivation of typical tumor suppressor genes (*TP53*, *NF1*, *PTEN*) allocates formation and early progression of high-grade astrocytoma to neural stem/progenitor cells within the SVZ [28–30]. In humans, intraoperative fluorescence-guided multiple sampling (FGMS) along a spatial gradient within the tumor mass and the adjacent (fluorescent) SVZ recently allowed for a phylogenetic reconstruction with SVZ-derived tumor precursor cells giving rise to the tumor mass in the majority of GBM patients analyzed, and thus, for the first time, substantiated a role of the SVZ in gliomagenesis in humans [16]. These observations also suggest that SVZ+ GBMs are enriched in NSCs and BTICs, a hypothesis that has not yet been explored in much detail. In a microarray analysis of 47 GBMs, Kappadakunnel et al*.* did not find a correlation between 7 selected stem cell-related genes of interest (*PROM1*, *MELK*, *BMP4*, *ETF2*, *MAPK8*, *OLIG2*, *NES*) and tumor location [15]. Instead, 7 of the 33 genes overexpressed in group II tumors were related to immune signaling (*FCGR3A*, *HLA-DRB5*, *BCL6*, *FCGR3B*, *MAFB*, *HLA-DRA*, *HLA-E*), a finding which was underscored by our MetaCore™ pathway analysis.

To further investigate a potential stem cell origin of SVZ+ GMB, we searched our microarray data for location-specific differential expression of a panel of 47 well-known NSC, BTIC, and lineage markers as well as key players of three development pathways (Notch, Wnt, and Sonic Hedgehog). Since group I GBMs that are defined as SVZ+ GBMs consist of voluminous tumors that reach from the cortical surface to the SVZ and, in theory, may reflect tumors that originate from the cortex rather than the SVZ, we decided to compare group II and group III tumors only, since this reflects the most concise regional separation between GBMs with (group II) and without (group III) SVZ contact. Differential regulation of gene expression was observed for a subset of genes only (*FZD6*, *PTCH2*, *NEFH*, *NOTCH2*, *HES4*, *DLL3*), including three genes (*NOTCH2*, *HES4*, *DLL3*) involved in Notch signaling. Remarkably, location-dependent differential gene expression was detected at all key hierarchies of the Notch pathway (depicted in Fig. 4). It is well known that Notch signaling drives NSC maintenance and differentiation of neural progenitor cells into astroglia (reviewed in [27]). Moreover, aberrant Notch pathway activation contributes to formation and propagation of primary GBM [31], possibly through propagation of the BTIC pool [32], and therapeutic inhibition of Notch signaling is under investigation both in vitro and in vivo (reviewed in [33]). In this regard, our study provides the first link between Notch expression and tumor location. With the Notch ligand DLL3 and the transcription factor HES4 we found pivotal upregulated genes that later proved to be prognostic in a multivariate setting. However, our data are restricted to the transcriptional level, and functional analyses are needed to shed further light on location-specific Notch pathway activation. Nevertheless, neither this analysis nor that of Kappadakunnel et al*.* found unequivocal evidence of a (cancer) stem cell signature in SVZ+ GBM [15]. This may account for the fact that differential expression was assessed on a transcriptional level only and that in-depth analysis of a larger patient sample, in particular combined with investigation of protein expression, may yield unambiguous results. A sampling error may also come into play. Since all samples were retrospectively identified from our tumor bank, it is impossible to reconstruct the exact position of sampling, in particular with regard to distance from the SVZ. Particularly in group I GBM, the most voluminous location-specific subgroup extending from the SVZ throughout the white matter to cortical areas, there may well be a spatial gradient in the enrichment of tumor tissue with NSCs and BTICs, a factor impossible to take into account unless tissue sampling is done in a prospective manner with multiple samples derived from the same tumor and the regions of interest defined on perioperative imaging [16, 34]. Ultimately, it would be worthwhile to compare transcriptomes from SVZ+ and SVZ– GBMs to those derived from non-malignant human SVZ to elucidate the role of the SVZ in human gliomagenesis. However, to the best of our knowledge, array data from human SVZ tissue have been lacking until now.

To attenuate the sampling issue, we ensured that all GBM tissues analyzed were homogeneous throughout the location groups in terms of tumor content and interplay from the microenvironment (Additional file 1: Figure S1A–D) and validated *HES4* and *DLL3* expression together with 11 other top differentially expressed candidate genes derived from microarray analysis in an independent set of 142 *IDH1* wild-type GBMs (validation cohort). Hence, we were able to confirm SVZ-dependent expression of *HES4* and *DLL3. HES4* was significantly overexpressed in both SVZ+ GBMs and group II GBMs. *HES4* is one of seven *HES* target genes of Notch signaling that serve as tissue-specific transcription repressors upon Notch pathway activation, leading to inhibition of cell differentiation and maintenance of stem cell features [35]. Little is known about the particular function of *HES4* in stem cells and cancer. *HES4* was shown to keep retinal precursor cells of the Xenopus ciliary margin zone in an undifferentiated and slowly proliferative state [36]. In human B cells, *HES4* inhibits early differentiation and acts as a tumor suppressor with epigenetic silencing in B cell acute lymphoblastic leukemia [37]. Recently, *HES4* has been established as a biomarker in advanced solid tumors, predicting treatment response to Notch pathway inhibition by gamma-secretase inhibitors [38, 39]. Notably, our analysis constitutes the first report on *HES4* interaction in human brain tumors.

*DLL3* is the second Notch pathway key player for which a strong trend towards overexpression in group II GBMs was confirmed in our analysis. *DLL3* is a direct ligand to Notch receptors, with conflicting data about its activating or rather inhibiting role in Notch signaling [40–44]. Likewise, *DLL3* has been described in the context of proneural GBM with inconsistent expression patterns: according to Phillips et al*.* [7] and Verhaak et al. [8], overexpression of *DLL3* is a hallmark of proneural GBM, while Cooper et al. [45] report loss of *DLL3* in proneural GBM. In the meantime, identification of an *IDH*-dependent G-CIMP phenotype has further separated the initially favorable appraised proneural molecular subgroup into GBMs with an *IDH*-mutant, G-CIMP-positive phenotype exhibiting a highly favorable prognosis and an *IDH*-wild-type, non-G-CIMP phenotype with an exceedingly dismal prognosis [9]. This is of importance, since none of the aforementioned studies stratified expression and survival data for *IDH* mutation status, while our study comprised *IDH1 wt* GBM only. Indeed, *DLL3* was significantly overexpressed in the 10 tumor samples of our microarray cohort assigned to the proneural subtype (*p* = 0.0078; Mann-Whitney test; data not shown), but no difference in OS was observed between proneural and other subtypes (*p* = 0.4776; log-rank test; data not shown).

We also observed a strong trend towards upregulation of *NTRK2* and downregulation of *PIR* in group II GBMs (contacting the SVZ only). *NTRK2* encodes for the neurotrophic tyrosine kinase, receptor, type 2, also known as Trk-B. Neurotrophins and their receptors are crucial for cell growth, survival, and apoptosis in the nervous system [46], but expression has been observed in glioma as well [47], even though their functional role is largely unknown. Activation of Trk-B and Trk-C has been shown to promote growth and survival of BTICs independent of epidermal growth factor (EGF) and basic fibroblast growth factor (bFGF) [48]. In our study, *NTRK2* overexpression was found in GBMs with SVZ contact, possibly maintaining BTIC growth as well. *PIR* is another location-specific candidate gene with downregulation observed in group II GBMs. It encodes for the iron-binding nuclear protein pirin, a transcriptional regulator, and has been described as an oncogene [49] and promoter of metastatic tumor growth [50] on one hand and as a tumor suppressor gene on the other [51] in many solid cancers, but never before in glioma. In acute myeloid leukemia (AML), *PIR* was linked to terminal differentiation of myeloid precursors with a downregulation of *PIR* possibly related to the differentiation arrest observed in AML [52]. By contrast, overexpression of *PIR* was involved in inhibition of cellular senescence in melanocytic cells, resulting in transformation to melanoma [53]. In GBM, the functional role of *PIR* has yet to be discovered.

Besides their SVZ-dependent expression, *HES4*, *DLL3*, *PIR*, and *NTRK2* also conferred a significant impact on patient survival, together with known clinico-pathological prognosticators. Importantly, the reported adverse effect of SVZ involvement on OS [13] was confirmed in our study sample by multivariate analyses. In line with its distinct expression in GBMs contacting the SVZ, *HES4* turned out to be the most robust prognostic marker with overexpression related to adverse OS, even overriding the prognostic effect of all other molecular markers in the multivariate analysis. As elucidated above, *HES4* is a novel molecular marker in GBM and underscores the biological and clinical role of Notch pathway activation in primary GBM, in particular in tumors involving the SVZ. Likewise, overexpression of *DLL3*, the second molecular marker involved in Notch signaling, resulted in significantly reduced OS and PFS in the univariate analysis and turned out to be an independent prognostic marker of inferior PFS in the multivariate analysis. Moreover, high expression of *PIR* and *NTRK2* was associated with superior OS. It is noteworthy that in patients with GBMs contacting the SVZ (SVZ+ GBMs, group II GBMs), *NTRK2* expression levels were able to predict OS. The beneficial effect of *NTRK2* overexpression on OS confirms recent data reporting that loss of mRNA expression of both *NTRK1* and *NTRK2* correlates with poor prognosis in patients with high-grade glioma [54], but our analysis adds a location-specific link to the picture. To our knowledge, this is the first report on *PIR* as a prognostic marker in GBM, with evidence of SVZ-dependent differential expression, and it is worth further functional analysis to investigate its role as an oncogenic or tumor repressive factor. In addition to these four genes with SVZ-dependent differential expression and concurrent prognostic impact, we also identified *IGFBP5* expression to be inversely related to OS, even though a location-specific expression could not be established. There is sparse evidence from the literature that overexpression of *IGFBP5* increases in a WHO grade-dependent fashion with highest expression observed in GBM [55, 56] and a strong trend linking overexpression to adverse OS [55]. The functional role of *IGFBP5* has not been fully clarified, but it may play a role in tumor dormancy, among others in GBM [57].

## Conclusions

In summary, this study revealed inherent transcriptional differences of GBMs, depending on their vicinity to the SVZ. Interestingly, all four genes with simultaneous SVZ-dependent differential expression and significant prognostic impact were characterized by their involvement in stem cell maintenance. Two of these genes (*HES4*, *PIR*) have never before been reported in the context of gliomagenesis and deserve further functional exploration. Importantly, Notch signaling was an outstanding feature of SVZ+ GBM, with the two key players *HES4* and *DLL3* identified as location-specific prognosticators. Further work will be required, but this finding suggests that SVZ+ GBM might profit most from the therapeutic Notch inhibition that is currently under investigation in clinical trials. Mounting evidence is in favor of location-tailored therapies, since irradiation of the ipsilateral SVZ as a potential BTIC niche has been shown to have a positive impact on patient outcome [17, 18], particularly in patients with GTR [19]. SVZ-specific targeted molecular therapies might add another important piece to the picture.

## Additional files

Additional file 1: Figure S1. Comparative analysis of the cellular composition of location-specific groups I–IV in the microarray discovery set (*n* = 36 GBMs). ANOVA analyses revealed no significant difference in all four comparisons. Difference of (A) tumor content determined by neuropathologist in hematoxylin and eosin (*HE*) stain, (B) StromalScore, (C) ImmuneScore, and (D) ESTIMATEScore between location groups. StromalScore, ImmuneScore, and ESTIMATEScore were calculated using the estimate package for the *R* software environment (Kosuke Yoshihara, Hoon Kim, and Roel GW Verhaak (2013), *ESTIMATE* Estimate of STromal and Immune cells in MAlignant Tumor tissues using Expression data, *R* package version 1.0.11). (DOCX 70 kb)
Additional file 2: Supplemental Methods. (DOCX 85 kb)
Additional file 3: Primers (obtained from SIGMA-Aldrich (St. Louis, USA)) and corresponding probes (designed with the Universal Probe Library Assay Design Center (http://lifescience.roche.com)) which were applied for quantitative PCR. (PDF 41 kb)

## Abbreviations

AML: Acute myeloid leukemia
BTIC: Brain tumor-initiating cells
CE: Contrast-enhancing
DLL3: Delta-like 3
EOR: Extent of resection
FGMS: Fluorescence-guided multiple sampling
GBM: Glioblastoma
G-CIMP: Glioma-CpG island methylator phenotype
GTR: Gross total resection
HES4: Hairy and enhancer of split 4
IDH1: Isocitrate dehydrogenase 1
IGFBP5: Insulin-like growth factor-binding protein 5
MGMT: *O* ^6^-methylguanine-DNA methyltransferase
mt: Mutant
NSC: Neural stem cell
NTRK2: Neurotrophic receptor tyrosine kinase, type 2
OS: Overall survival
PFS: Progression-free survival
PIR: Pirin
qPCR: Quantitative polymerase chain reaction
SVZ: Subventricular zone
TCGA: The Cancer Genome Atlas
TMZ: Temozolomide
WHO: World Health Organization
wt: Wild-type
