# Supplementary material for: Spatial transcriptome analysis reveals Notch pathway-associated prognostic markers in IDH1 wild-type glioblastoma involving the subventricular zone
Source: BMC Med. 2016 Oct 26;14:170. doi: 10.1186/s12916-016-0710-7 (PMC5080721; doi:10.1186/s12916-016-0710-7)
Supplement: Additional file 1: Figure S1. — Comparative analysis of the cellular composition of location-specific groups I–IV in the microarray discovery set (n = 36 GBMs). ANOVA analyses revealed no significant difference in all four comparisons. Difference of (A) tumor content determined by neuropathologist in hematoxylin and eosin (HE) stain, (B) StromalScore, (C) ImmuneScore, and (D) ESTIMATEScore between location groups. StromalScore, ImmuneScore, and ESTIMATEScore were calculated using the estimate package for the R software environment (Kosuke Yoshihara, Hoon Kim, and Roel GW Verhaak (2013), ESTIMATE Estimate of STromal and Immune cells in MAlignant Tumor tissues using Expression data, R package version 1.0.11). (DOCX 70 kb) [file 12916_2016_710_MOESM1_ESM.docx]

**Additional file 1**

**Figure S1** Comparative analysis of the cellular composition of location-specific groups I-IV in the microarray discovery set (n=36 GBM). ANOVA analyses revealed no significant difference in all four comparisons. Difference of **(A)** tumor content determined by neuropathologist in hematoxylin and eosin (HE) stain, **(B)** StromalScore, **(C)** ImmuneScore and **(D)** ESTIMATEScore between location groups. StromalScore, ImmuneScore and ESTIMATEScore were calculated using the estimate package for the R software environment (Kosuke Yoshihara, Hoon Kim and Roel GW Verhaak (2013). estimate: ESTIMATE: Estimate of STromal and Immune cells in MAlignant Tumor tissues using Expression data. R package version 1.0.11.).
