# Supplementary material for: Spatial transcriptome analysis reveals Notch pathway-associated prognostic markers in IDH1 wild-type glioblastoma involving the subventricular zone
Source: BMC Med. 2016 Oct 26;14:170. doi: 10.1186/s12916-016-0710-7 (PMC5080721; doi:10.1186/s12916-016-0710-7)
Supplement: Additional file 2: — Supplemental Methods. (DOCX 85 kb) [file 12916_2016_710_MOESM2_ESM.docx]

**Supplemental Methods**

**Quality Control of GBM Tissues**

We applied rigorous inclusion criteria of at least 60% tumor content and less than 20% necrosis in the hematoxylin and eosin stain for all tumor tissues analyzed. Comparing the distribution of tumor content between the four location-specific groups I-IV did not reveal a significant difference (**Figure S1A**). Next, we aimed to digitally deconvolute the tumor microenvironment and interrogate possible location-dependent differences. To this end, we applied the ESTIMATE algorithm by Yoshihara et al. (1). Employing single-sample gene set-enrichment analysis in a large patient cohort, the authors developed a StromalScore, ImmuneScore and the ESTIMATEScore as a combination of StromalScore and ImmuneScore inferring tumor purity. Applying the estimate R package (Kosuke Yoshihara, Hoon Kim and Roel GW Verhaak (2013). estimate: ESTIMATE: Estimate of STromal and Immune cells in MAlignant Tumor tissues using Expression data. R package version 1.0.11.) to our microarray discovery set (n=36 GBM) revealed no significant difference between the four location groups for any of the three scores (**Figure S1B-D**). Hence, at least on the transcriptional level we presume that the changes observed between SVZ+ vs. SVZ- GBM and group II vs. group III GBM are not significantly driven by differences in cell admixture.

**Reference**

1. Yoshihara K, Shahmoradgoli M, Martínez E, Vegesna R, Kim H, Torres-Garcia W, et al. Inferring tumour purity and stromal and immune cell admixture from expression data. Nat Commun. 2013 Oct 11;4:2612.
