# Supplementary material for: Spatial transcriptome analysis reveals Notch pathway-associated prognostic markers in IDH1 wild-type glioblastoma involving the subventricular zone
Source: BMC Med. 2016 Oct 26;14:170. doi: 10.1186/s12916-016-0710-7 (PMC5080721; doi:10.1186/s12916-016-0710-7)
Supplement: Additional file 3: — Primers (obtained from SIGMA-Aldrich (St. Louis, USA)) and corresponding probes (designed with the Universal Probe Library Assay Design Center (http://lifescience.roche.com)) which were applied for quantitative PCR. (PDF 41 kb) [file 12916_2016_710_MOESM3_ESM.pdf]

SUPPL. TABLE 1

Gene	Forward Primer (5' > 3')	Reverse Primer (3' > 5')	Probe
**PIR**	TCCTGATCATCCACATCGAG	TTACCAGTGTGTCCACAGAAGTC	79
**IGFBP5**	AGAGCTACCGCGAGCAAGT	GTAGGTCTCCTCGGCCATCT	77
**HES4**	CTGGACGCCCTCAGAAAA	GCTCCGCAGGTGTCTCAC	80
**DLL3**	CAACTGTGAGAAGAGGGTGGA	CCAGGTCCAGGCAGAGTC	46
**NTRK2**	GGTTTGGATTTGGGAAAGTAAA	GGCAGAGTCATCATCATTGC	75
**BAI3**	TGTAATATTGCTCTTTGCCCAGT	CCATTCGAGCACGTTACTGA	36
**EMILIN3**	CCCCTTCCTACAGCAGAGC	GCTGGACATCACCCTCCA	23
**PDGFRA**	CCACCTGAGTGAGATTGTGG	TCTTCAGGAAGTCCAGGGAA	27
**FERMT2**	TGAAGTTGATGCTGCCCTTT	TCAGGAATGGAAGTAATGTCACC	85
**CDH4**	CAGACCCCGTAACCAACG	TGAAAGCTCTGTTGAGCTCGT	8
**UCHL1**	AGATCAACCCCGAGATGCT	ACCGAGCCCAGAGACTCC	45
**HIF1A**	GATAGCAAGACTTTCCTCAGTCG	TGGCTCATATCCCATCAATTC	64
**TGFB3**	AAGAAGCGGGCTTTGGAC	CGCACACAGCAGTTCTCC	38
**RBP1**	AGGCATAGATGACCGCAAGT	ACCCTTCTGCACACACTGG	33
**SYTL4**	CCAGAAGCTAGCCAGAAGCA	TCATGCTGCCGATCGTACT	25
**THBS4**	CCTGAGACCATTGAATTGAGG	ACCAGCTTCAGCTCTTCCAA	6
**NODAL**	GGCGAGTGTCCTAATCCTGT	GCTGGTAACGTTTCAGCAGACT	52
**EPHB1**	TCTCCAGCAAGAGTCCCTTC	GCACTGACTTGGTGCATGAT	9
**FZD6**	TTGGCATCTCTGCTGTCTTC	TCTTCGACTTTCACTGATTGGA	23
**VAV3**	CCTTAGATACAACTCTGCAGTTTCC	GCCCAGCACTTTTGGACTTA	36
**ENPP5**	TGGATAAAGACCACTATACCCTGAT	GCTTCATAGACTTCATCAAATTTACCT	46
**NDN**	CTGATGATGTGTGTTGGGGTA	GGCTTTGCTGGTGACTTCTT	68
**NFKBIA**	GTCAAGGAGCTGCAGGAGAT	ATGGCCAAGTGCAGGAAC	38
**IRF9**	ACCAGGATGCTGCCTTCTT	TCCTGTGTCCCCCTCCTTAT	61
**BATF3**	CAGCGTCCTGCAGAGGAG	CTTCGGACCTTCCTGTCATC	28
**ANGPTL2**	GTGGACCCTGAGGTCCTTC	CCACCTTGTGGAAGAGTTGC	18
**PROM1**	TCCACAGAAATTTACCTACATTGG	CAGCAGAGAGCAGATGACCA	83
**FABP7**	GAAATTAAGGATGGCAAAATGG	CTCATAGTGGCGAACAGCAA	9
**GAPDH**	AGCCACATCGCTCAGACAC	GCCCAATACGACCAAATCC	60
**ACTB**	CCAACCGCGAGAAGATGA	CCAGAGGCGTACAGGGATAG	64
**HPRT1**	TGACCTTGATTTATTTTGCATACC	CGAGCAAGACGTTCAGTCCT	73
